# Intrathoracic Omental Hernia Repaired by Laparoscopy: A Case Report

**DOI:** 10.7759/cureus.85838

**Published:** 2025-06-12

**Authors:** Akitsugu Fujita, Masaya Watanabe, Masato Nishida, Shinsuke Sato, Ko Ohata, Hideyuki Kanemoto

**Affiliations:** 1 Gastroenterological Surgery, Shizuoka General Hospital, Shizuoka, JPN

**Keywords:** esophageal hiatal hernia, intrathoracic omental hernia, laparoscopic hernia repair, laparoscopic surgery, type-ii hiatal hernia

## Abstract

Intrathoracic omental hernia (ITOH) is a rare disease with only 17 previously reported cases. ITOH is a hiatal hernia in which the hernia content is only greater omentum, but the cause of the onset is unknown. There have been reports of ITOH requiring emergency surgery due to intussusception during follow-up, and surgery is recommended even if the patient is asymptomatic. The patient is a 79-year-old man. He visited his local doctor with a chief complaint of chest tightness during swallowing. Chest X-ray revealed a mediastinal tumor, and he was referred to our hospital. He was diagnosed with ITOH by MRI and contrast-enhanced CT scan. He was symptomatic and underwent laparoscopic hernia repair. Intraoperative findings revealed a greater omental prolapse into the posterior mediastinum from the right side of the esophageal hiatus. The greater omentum was returned manually into the abdominal cavity, and the hernial orifice was sutured closed with a 3-0 barbed suture. The patient had a good postoperative course and was discharged home on the sixth postoperative day. Laparoscopic surgery for ITOH was considered to be an effective treatment method because of its low invasiveness and easy repair of the hernial orifice.

## Introduction

Intrathoracic omental hernia (ITOH) is a type of hiatal hernia of the esophagus. In esophageal hiatal hernia, the stomach usually escapes into the mediastinum through the esophageal hiatus, but ITOH is a rare condition in which there is no displacement of the stomach and only the omentum escapes into the mediastinum through the esophageal hiatus. The cause of ITOH is unknown. In some previous reports, open thoracotomy was performed because of difficulty in differentiating ITOH from liposarcoma in the mediastinum in the preoperative diagnosis. In this report, we describe a case in which ITOH was diagnosed preoperatively and repaired laparoscopically with good results.

## Case presentation

**Figure 1:**
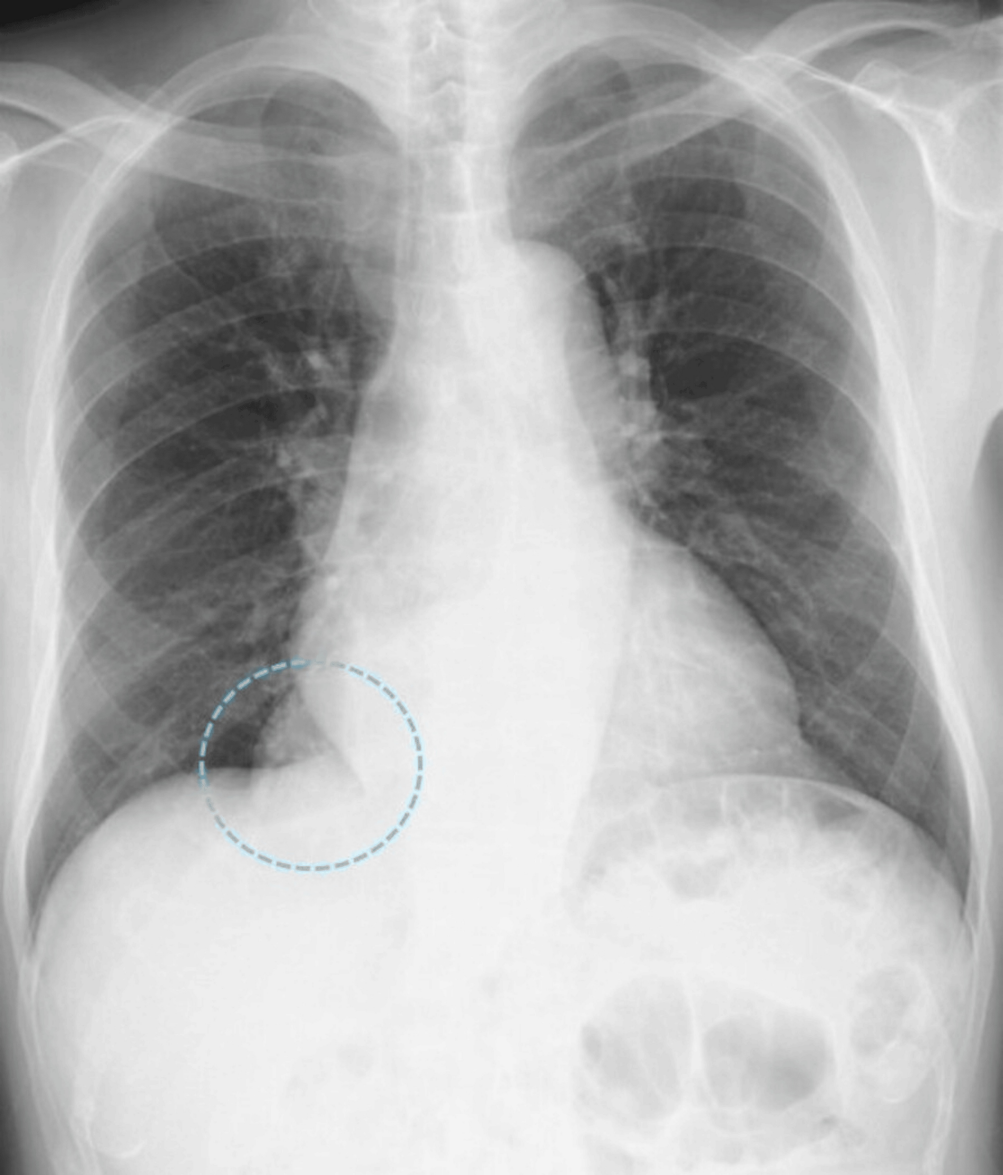
Chest X-ray Abnormal mediastinal shadows were noted, and a mediastinal tumor was suspected

**Figure 2:**
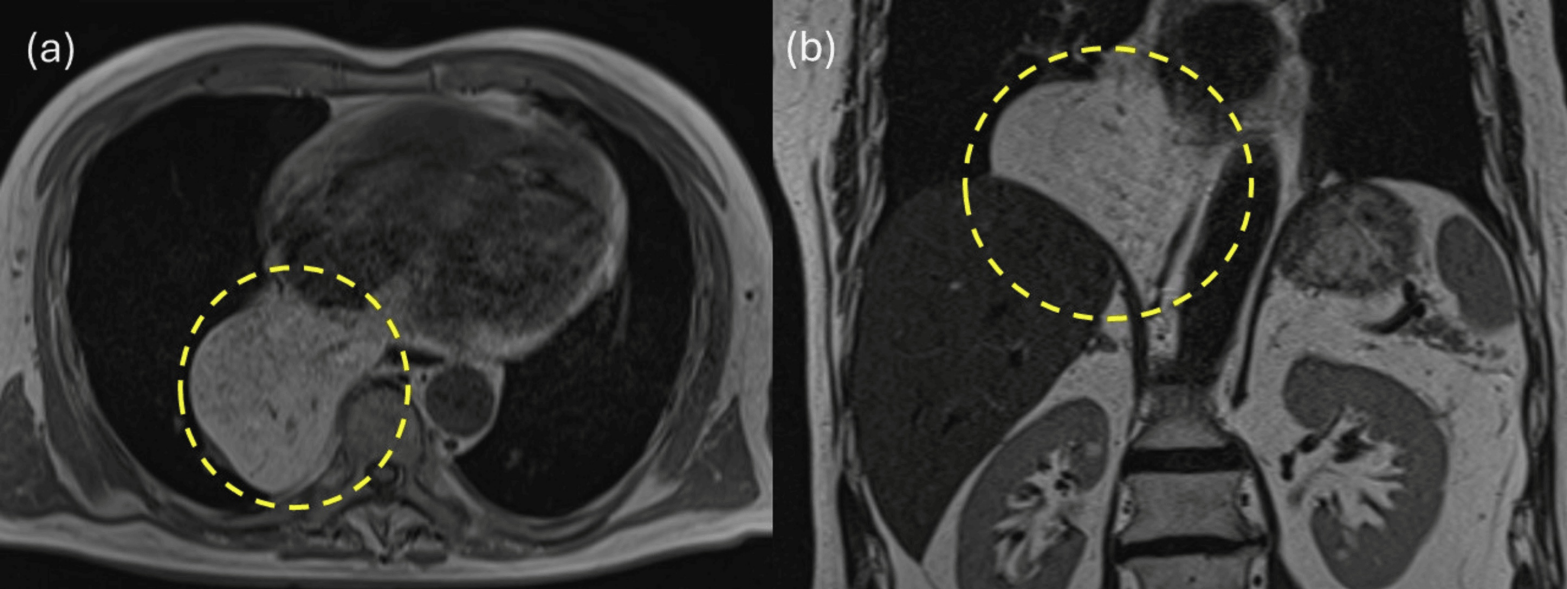
Contrast-enhanced MRI Prolapse of the intraabdominal fat into the mediastinum via the esophageal hiatus

**Figure 3:**
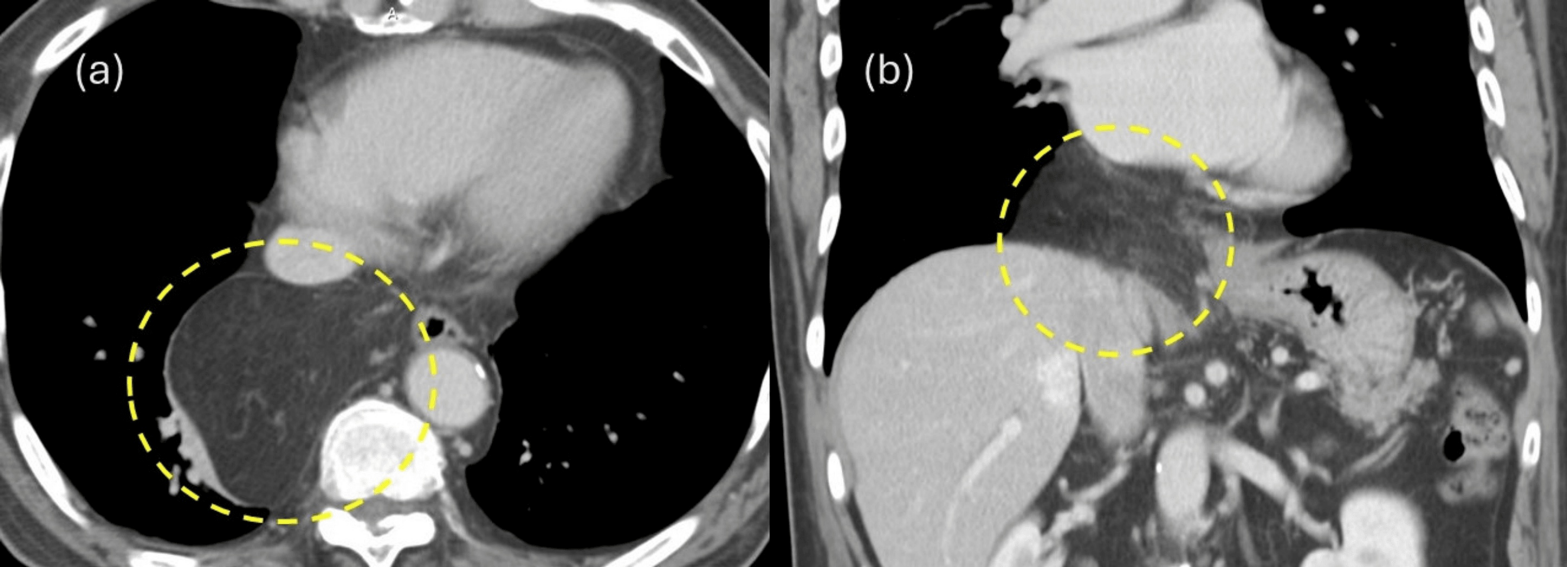
Contrast-enhanced CT Intraabdominal fat was found to be prolapsed into the mediastinum via the esophageal hiatus

**Figure 4:**
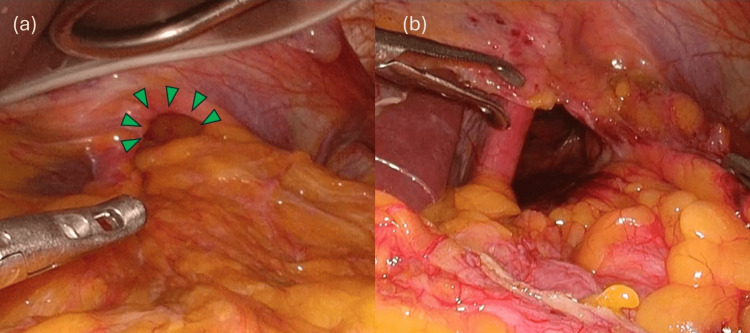
Surgical observations (a) Prolapse of the greater omentum into the mediastinum via the esophageal hiatus. (b) Returning the greater omentum that had fallen into the mediastinum into the abdominal cavity

A 79-year-old man presented to his local doctor for chest tightness that had been present for approximately one month. This patient was 169.7 cm tall and weighed 70.2 kg. An electrocardiogram showed a heart rate of 65 beats per minute and sinus rhythm. Physical examination revealed no abnormalities in the chest or abdomen. Blood test results also showed no abnormalities. The patient had no history of gastrointestinal or cardiovascular disease, and no history of abdominal or chest trauma was noted during the medical interview. However, the patient had a history of diabetes and dyslipidemia and was taking medication regularly. A chest X-ray revealed a suspected mediastinal tumor (Figure 1), and the patient was referred to a respiratory physician. After additional contrast-enhanced MRI (Figure 2) and CT (Figure 3), ITOH was suspected due to the extension of blood vessels in the greater omentum into the mediastinum via the esophageal hiatus, and the patient was referred to the gastrointestinal surgery department. The patient underwent laparoscopic hernia repair for a symptomatic hernia. Intraoperative findings showed that the right side of the esophageal hiatus was the hernial orifice (Figure 4a), and the greater omentum was prolapsing into the right posterior mediastinum. The greater omentum was not adherent to the hernia sac and could be reduced manually into the abdominal cavity (Figure 4b).

**Figure 5:**
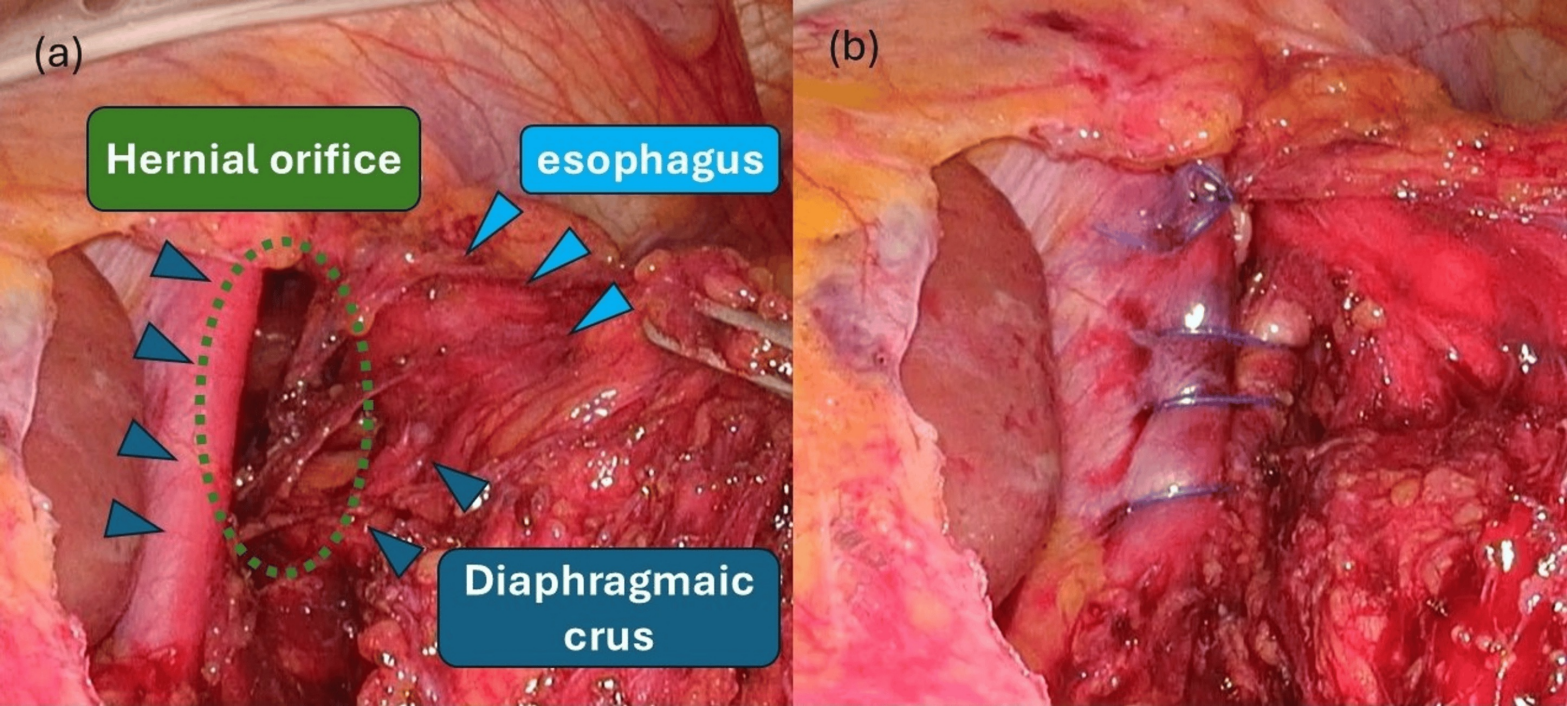
Surgical observations Left and right diaphragmatic legs sutured with 3-0 barbed suture thread to close the hernial orifice

The hernial orifice was an esophageal hiatus (Figure 5a), but there was no gastric prolapse, and the only hernia content was a greater omentum. After returning the greater omentum into the abdominal cavity, the right and left crus of the diaphragm were sutured with continuous nonabsorbable 3-0 barbed suture, and the hernial orifice was closed (Figure 5b).

The operation time was two hours and 19 minutes, and blood loss was minimal. The postoperative course was uneventful, and the patient was discharged home on the sixth postoperative day. There has been no recurrence for one year after surgery.

## Discussion

Esophageal hiatal hernia is a diaphragmatic hernia in which organs in the abdominal cavity prolapse into the thoracic cavity through the esophageal hiatus. Most esophageal hiatal hernias are of the prolapsed type, in which a portion of the stomach prolapses toward the thoracic cavity, often resulting in symptoms such as acid regurgitation due to gastric acid reflux. This is often acquired due to age-related weakening of the transverse esophageal ligament or increased intrathoracic pressure caused by obesity. In the present case, only the greater omentum was prolapsed, which is called ITOH [1]. ITOH is a rare disease, and only 18 cases of surgery for ITOH have been reported in PubMed. Only seven of the reported cases were repaired laparoscopically (Table 1), including our case, and are considered to be rare [1-6].

**Table 1:** Eighteen surgical cases of ITOH ITOH: intrathoracic omental hernia There are only 18 reports of surgery for ITOH, of which only seven cases, including this case, have been reported with laparoscopic hernia repair

Case	Year	Author	Aage	Gender	Symptoms	Size of hernial orifice	Treatment	Repair methods
1	1966	Pomerantz [7]	55	M	Chest tightness	Unknown	Thoracotomy	Unknown
2	1977	Rohlfing [8]	50	M	Abdominal pain	Unknown	Open surgery	Unknown
3	1988	Tamura [9]	56	F	None	Unknown	Thoracotomy	Unknown
4	1993	Rockoff [10]	67	M	None	Unknown	Open surgery	Unknown
5	1999	Kato [11]	54	F	None	Unknown	Thoracotomy	Unknown
6	1999	Anderson [12]	43	M	Difficulty in swallowing	Unknown	Thoracotomy	Unknown
7	2004	Yunoki [13]	61	M	None	Unknown	Thoracotomy	Unknown
8	2005	Maruyama [14]	21	M	None	Unknown	Thoracotomy	Unknown
9	2013	Stephens [4]	61	M	Abdominal pain	Unknown	Laparoscopy	Unknown
10	2013	Yu [15]	59	M	None	Unknown	Thoracotomy	Unknown
11	2017	Zhu [16]	47	M	Chest tightness	Unknown	Thoracotomy	Unknown
12	2017	Sueyoshi [5]	46	M	Abdominal pain	Unknown	Laparoscopy	Direct closure
13	2019	Wang [17]	47	M	Dyspnea	Unknown	Thoracotomy	Unknown
14	2020	Tanaka [6]	60	M	Abdominal pain	5 ㎝	Laparoscopy	Unknown
15	2021	Nakashima [1]	72	M	None	Unknown	Laparoscopy	Direct closure
16	2021	Rassam [3]	63	M	None	3 ㎝	Laparoscopy	Mesh use
17	2022	Futawatari [2]	53	F	Heartburn	5 ㎝	Laparoscopy	Direct closure
18	2024	Present case	79	M	Chest tightness	3 ㎝	Laparoscopy	Direct closure

ITOH is often asymptomatic and is often discovered when an enlarged cardiac shadow is noted on chest radiographs during medical examinations. In symptomatic cases, patients may present with chest discomfort, heartburn, and vomiting, as in the present case.

With the improvement of CT and MRI diagnostic imaging capabilities, ITOH can be diagnosed preoperatively by finding the　omentum escaping into the thoracic cavity through the esophageal hiatus before the operation. However, in several cases that were not diagnosed as ITOH by laparoscopic repair, it was difficult to distinguish ITOH from mediastinal liposarcoma and other tumors because it was difficult to diagnose ITOH preoperatively by CT or MRI imaging, and open resection was performed. The preoperative diagnosis of ITOH is to confirm by CT or MRI that the hernia contents in the mediastinum are fatty tissue and that the vessels within the prolapsed fatty tissue are continuous from the abdominal cavity via the esophageal hiatus. In some of the reported cases, the hernia contents were fitted, and emergency surgery was performed [17], so it is desirable to perform hernia repair prophylactically when ITOH is observed. As for the surgical method, there were no reports of recurrence in the seven patients who underwent laparoscopic repair, and in the autopsy case, there was no recurrence for one year after the surgery, suggesting that laparoscopic repair is useful. Direct suture closure of the hernial orifice was performed in four of the five cases in which the method of hernial orifice repair was described [1,2,4,5], and mesh was reported in only one case [3], but there were no reports of postoperative complications in any of the reports [18]. Considering the potential for complications from prolonged mesh retention, direct suture closure of the hernia orifice alone is considered sufficient. However, in esophageal hiatal hernia repair, mesh repair has a lower recurrence rate than direct suture repair, and there is no difference in complication rate [19]. In previous ITOH reports, there have been no reports of recurrence in either the mesh or direct suture group, but the number of cases is small, and further study is needed.

ITOH is considered to be a type of hiatal hernia because of the prolapse of the greater omentum into the thoracic cavity via the esophageal hiatus. However, the usual sliding hiatal hernia is caused by a deviation of the esophagogastric junction due to relaxation of the phrenoesophageal membrane with age or abdominal pressure [4]. The pathogenesis of ITOH is assumed to be similar to that of type-II hiatal hernia, in which the esophagogastric junction is not displaced. The pathogenesis of type-II hiatal hernia has not been reported yet, and we hope that more cases will be accumulated in the future to investigate the pathogenesis and preventive measures against it.

## Conclusions

We experienced a case of intrathoracic omental hernia that was repaired laparoscopically. When preoperative CT and MRI showed evidence of omental prolapse into the thoracic cavity via the esophageal hiatus, a diagnosis of ITOH is made, and prophylactic hernia repair is recommended. Laparoscopic hernia repair is considered to be useful in the treatment of this disease.
